# Investigando o Papel Causal das Citocinas Inflamatórias no Desenvolvimento de Doenças Cardiovasculares, Renais e Metabólicas

**DOI:** 10.36660/abc.20250611

**Published:** 2026-05-26

**Authors:** Mengjin Hu, Zhaoting Gong, Xiaosong Li, Chunlin Yin

**Affiliations:** 1 Xuanwu Hospital Capital Medical University Beijing China Xuanwu Hospital, Capital Medical University, Beijing – China; 2 Beijing Anzhen Hospital Capital Medical University Beijing China Beijing Anzhen Hospital, Capital Medical University, Beijing – China

**Keywords:** Citocinas, Inflamação, Doenças Cardiovasculares, Insuficiência Renal Crônica, Análise da Randomização Mendeliana, Interleucina-6

## Abstract

**Fundamento:**

Estudos observacionais têm relacionado citocinas inflamatórias a distúrbios cardiometabólicos; no entanto, ainda não está claro se essas associações refletem efeitos causais em sistemas orgânicos interconectados.

**Objetivos:**

Este estudo de randomização mendeliana (RM) investiga a causalidade entre 91 citocinas inflamatórias e disfunção multiorgânica nos sistemas cardiovascular, renal e metabólico na era da síndrome cardiovascular-renal-metabólica (CKM).

**Métodos:**

Foram obtidos instrumentos genéticos associados a 91 citocinas inflamatórias do painel Olink Target Inflammation em 11 grupos de ascendência europeia.A método da foi considerado a principal análise, complementadas por análises de sensibilidade, incluindo os métodos MR-Egger e da mediana ponderada.

**Resultados:**

Os níveis de citocinas inflamatórias previstos geneticamente mostraram amplas associações causais em diversas características relacionadas à matriz extracelular (CKM). Especificamente, 31 citocinas foram associadas a desfechos cardiovasculares, 26 a características renais, 10 a características relacionadas à obesidade e 21 a características relacionadas ao diabetes, totalizando 60 citocinas com pelo menos uma associação significativa. Notavelmente, FGF5 e IL6 emergiram como reguladores paradoxais, exercendo efeitos opostos em diferentes sistemas orgânicos, enquanto o MCSF foi consistentemente identificado como um amplificador de risco pan-vascular para doenças cardiovasculares.

**Conclusões:**

Esta análise abrangente de RM revela uma heterogeneidade causal substancial nos efeitos das citocinas inflamatórias nos sistemas cardiovascular, renal e metabólico. Esses achados destacam a importância clínica da modulação inflamatória específica para cada tecido e contexto, apoiando o desenvolvimento de estratégias terapêuticas de precisão para a síndrome de CKM.

## Graphical abstract

**Figura Central:**
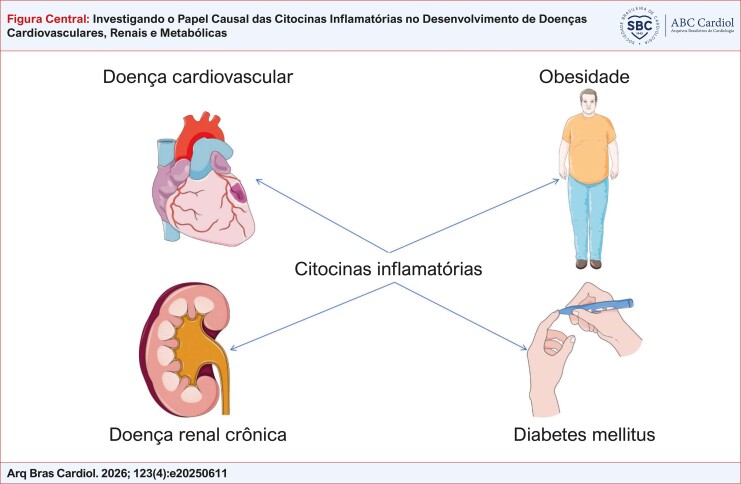
: Investigando o Papel Causal das Citocinas Inflamatórias no Desenvolvimento de Doenças Cardiovasculares, Renais e Metabólicas

## Introdução

Obesidade, diabetes e doença renal crônica (DRC) constituem uma tríade de condições epidemiologicamente interconectadas que, coletivamente, amplificam a morbidade e a mortalidade por doenças cardiovasculares (DCV) por meio de fisiopatologia sinérgica.^1^ Reconhecendo essas interações complexas, a American Heart Association estabeleceu recentemente a síndrome cardiovascular-renal-metabólica (CKM) como uma nova entidade diagnóstica – uma desordem multissistêmica caracterizada pela interação dinâmica entre desregulação metabólica, comprometimento cardiovascular e disfunção renal.^2^ É importante ressaltar que a síndrome CKM não é definida como uma única entidade patológica, mas sim como um espectro clínico que engloba obesidade, diabetes mellitus, DRC e suas sequelas cardiovasculares, incluindo insuficiência cardíaca (IC), fibrilação atrial (FA), doença arterial coronariana (DAC), acidente vascular cerebral (AVC) e doença arterial periférica (DAP).^2^ Essa conceitualização destaca as vias biológicas compartilhadas e a natureza progressiva da disfunção cardiometabólica e renal em todos os sistemas orgânicos. Em consonância com sua ampla relevância clínica, análises dos dados do National Health and Nutrition Examination Survey (NHANES) de 2011 a 2018 indicam que fatores de risco ou manifestações subclínicas (estágios 1 a 3) da CKM estão presentes em 80,94% dos adultos jovens (20 a 44 anos), 85,95% dos indivíduos de meia-idade (45 a 64 anos) e 72,03% da população idosa (≥65 anos).^3^

Do ponto de vista da pesquisa, no entanto, a síndrome CKM atualmente carece de um fenótipo genético unificado e validado, adequado para análises de inferência causal. Consequentemente, buscamos operacionalizar a CKM examinando individualmente características representativas dos sistemas cardiovascular, renal e metabólico, interpretando-as dentro de uma estrutura multissistêmica integrada. Essa abordagem baseada em características permite a avaliação sistemática de mecanismos compartilhados e divergentes entre os sistemas orgânicos e está alinhada com a compreensão contemporânea da CKM como um continuum em nível sistêmico, em vez de uma entidade nosológica singular.

Esclarecer os mecanismos fisiopatológicos subjacentes a essas doenças interconectadas é crucial para o desenvolvimento de estratégias terapêuticas direcionadas e para a melhoria dos resultados para os pacientes. Pesquisas recentes têm se concentrado cada vez mais nos mecanismos inflamatórios subjacentes que ligam essas doenças.^4,5^ No entanto, estabelecer relações causais definitivas continua sendo um desafio em pesquisas observacionais devido a limitações metodológicas persistentes, incluindo variáveis de confusão não previstas e causalidade reversa.

Avanços metodológicos recentes estabeleceram a Randomização Mendeliana (RM) como um paradigma analítico robusto para inferência causal em pesquisas epidemiológicas.^6-8^ Essa abordagem utiliza a segregação quase aleatória de variantes genéticas durante a gametogênese como mecanismo de randomização da natureza, permitindo estimativas causais que se aproximam de ensaios clínicos randomizados. Ao usar variantes genéticas como variáveis instrumentais, a RM^9^ pode mitigar efetivamente o viés de confusão e a causalidade reversa que frequentemente afetam estudos observacionais.^10^ A RM tem sido amplamente utilizada para investigar fatores de risco de doenças cardiovasculares e para avaliar os efeitos causais de intervenções terapêuticas.^9,11^ Neste estudo, extraímos inicialmente variantes genéticas válidas dos dados resumidos de estudos de associação genômica ampla (GWAS) publicados de 91 citocinas inflamatórias para investigar suas correlações comdoenças cardiovasculares, renais e metabólicas.

## Métodos

Nossa investigação de RM seguiu as diretrizes de relato STROBE-MR,^1 2^ atendendo a três pressupostos epidemiológicos fundamentais necessários para inferência causal válida: A) relevância do instrumento: variantes genéticas demonstram associações significativas em todo o genoma com a exposição; B) independência de fatores de confusão: variantes genéticas não estão relacionadas a nenhum fator de confusão na associação entre fator de risco e desfecho; C) restrição de exclusão: variantes genéticas não estão conectadas ao desfecho por meio de nenhuma via além do fator de risco de interesse. A visão geral do desenho do estudo é apresentada na Figura Suplementar 1.

### Fontes de dados

Os dados de GWAS de 91 citocinas inflamatórias foram quantificados usando o painel Olink Target Inflammation em 11 coortes de ascendência europeia (N=14.824).^1 3^ As análises de DCV incluíram IC (47.309 casos e 930.014 controles),^14^ FA (60.620 casos e 970.216 controles),^15^ DAC (CARDIoGRAMplusC4D Consortium, 60.801 casos e 123.504 controles),^16^ AVC (UK Biobank, 6.925 casos e 477.673 controles)^17^ e DAP (7.114 casos e 475.964 controles).^18^ As características de DRC incluíram DRC (coorte FinnGen, 3.902 casos e 212.841 controles), taxa de filtração glomerular estimada (TFGe, CKDGen Consortium e UK Biobank, 1.159.871 participantes),^19^ creatinina sérica (FinnGen e UK Biobank, 344.104 participantes),^18^ microalbuminúria (CKDGen Consortium, 54.116 participantes).^20^ Características de obesidade incluíram obesidade (UK Biobank, 4.688 casos e 458.322 controles), índice de massa corporal (IMC, 532.396 participantes),^21^ circunferência da cintura (CC, UK Biobank, 407.661 participantes).^22^ Características de diabetes incluíram diabetes tipo 2 (DM2, 61.714 casos e 1.178 controles),^23^ glicemia de jejum (200.622 participantes),^24^ e hemoglobina glicada A1c (HbA1c, UK Biobank, 389.889 participantes).^22^ Todas as análises utilizaram estatísticas sumárias disponíveis publicamente, com aprovação ética prévia e consentimento dos participantes obtidos em estudos originais por meio de comitês de revisão institucional, isentando Este estudo está sujeito a requisitos adicionais de revisão ética.

### Seleção de variáveis instrumentais

Inicialmente, o limiar de significância genômica (P<5×10^-8^) foi empregado para identificar associações fortespolimorfismos de nucleotídeo único (SNPs) com citocinas inflamatórias. Devido à disponibilidade limitada de instrumentos nesse limiar rigoroso, implementamos um critério de significância mais flexível (P<1×10^-5^).^-5^) mantendo um rigoroso controle de qualidade.^25^ Para garantir a independência genética, os procedimentos de agrupamento foram realizados com parâmetros definidos em um tamanho de janela de 10.000 kb e r^2^<0,001. A força do instrumento foi quantificada por meio de cálculos da estatística F, com variantes que demonstraram F>10 retidas para mitigar o viés de instrumentos fracos. A estatística F foi calculada usando a fórmula padrão $F=(\beta /\mathrm{se}{)}^{2}$ para cada instrumento genético. Finalmente, SNPs palindrômicos foram sistematicamente excluídos para manter a orientação consistente dos alelos em todos os conjuntos de dados de exposição-desfecho, evitando assim artefatos de desalinhamento de fita.

### Análise estatística

Os métodos de ponderação pela variância inversa (IVW), MR-Egger e mediana ponderada foram utilizados para inferir causalidade. O método IVW, que assume que todas as variantes genéticas são variáveis instrumentais válidas, foi considerado a análise principal. Na ausência de pleiotropia horizontal, o IVW pode fornecer uma estimativa causal não viesada.^26^ O método MR-Egger pode detectar e ajustar a pleiotropia direcional sob a suposição INSIDE, mas frequentemente com poder estatístico reduzido.^27^ A regressão MR-Egger foi utilizada para avaliar a pleiotropia horizontal. O intercepto diferente de zero da regressão MR-Egger sugeriu pleiotropia direcional.^27^ O método da mediana ponderada tem maior tolerância a variáveis instrumentais inválidas e ainda pode produzir estimativas confiáveis, desde que mais da metade do peso total provenha de variáveis instrumentais válidas.^28^ O teste Q de Cochran foi utilizado para avaliar a heterogeneidade entre as variáveis instrumentais. Um valor de p bicaudal < 0,05 foi considerado estatisticamente significativo para as análises primárias. Além disso, um limiar corrigido de Bonferroni de p < 0,003 (α = 0,05/15 desfechos) foi aplicado para levar em consideração as múltiplas comparações. Todas as análises de RM foram realizadas no software R (versão 4.4.1), utilizando o pacote “TwoSampleMR” (versão 0.6.8).

## Resultados

### Influência de 91 citocinas inflamatórias em características de DCV

**Figura 1:**
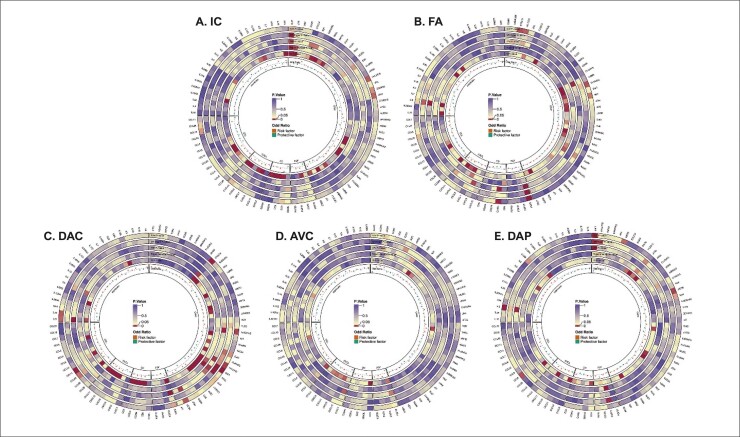
– Resultados do mapa de calor anular das 91 citocinas inflamatórias sobre características de DCV (Doenças Cardiovasculares). FA: fibrilação atrial; DAC: doença arterial coronariana; DCV: doença cardiovascular; IC: insuficiência cardíaca; DAP: doença arterial periférica.

A Figura 1 descreve o mapa de calor anular que representa 91 citocinas em doenças cardiovasculares, com análises IVW revelando arquiteturas de risco distintas. Níveis geneticamente elevados de CCL28, CD5, CXCL10, HGF e IL18R1 (aumento de um desvio padrão) foram associados a um risco aumentado de IC (Figura 1A), enquanto CCL19, LIFR, SULT1A1 e TRANCE demonstraram efeitos protetores. Para FA (Figura 1B), o FGF5 exibiu amplificação de risco, contrastando com as associações protetoras para CCL25, CD274, CD40L, FLT3LG, IL6, SULT1A1, TNFSF12 e uPA. As análises de DAC identificaram CX3CL1, IL10, IL6, LIFR, NRTN, OPG, SCF e SULT1A1 como mediadores protetores, enquanto CCL11, CCL20, EIF4EBP1, FGF5, FLT3LG e MCSF emergiram como potentes fatores aterogênicos (Figura 1C). O risco de AVC foi amplificado por FGF21 e MCSF (Figura 1D), enquanto a DAP exibiu regulação divergente, com IL6 conferindo proteção apesar dos efeitos persistentes de MCSF no risco (Figura 1E). As análises de sensibilidade entre os métodos MR-Egger e de mediana ponderada validaram essas associações (Figura Suplementar 2), destacandoO MCSF como um amplificador de risco pan-vascular para DCV. Entre as numerosas associações significativas identificadas, várias permaneceram significativas apósBonferroniCorreção. FGF5 (odd ratio [OR] = 1,065, intervalo de confiança [IC] de 95%: 1,036–1,096; P < 0,001) foi associado a um risco aumentado de FA, CCL11 (OR = 1,085; IC de 95%: 1,028–1,144; P = 0,003) e FLT3LG (OR = 1,087; IC de 95%: 1,029–1,150; P = 0,003) foram associados a um risco aumentado de DAC, enquanto LIFR (OR = 0,889; IC de 95%: 0,832–0,951; P = 0,001) foi associado a um risco diminuído de DAC. IL6 (OR=0,834; IC 95%: 0,740–0,939; P=0,003) e LIFR (OR=0,876; IC 95%: 0,809–0,949; P=0,001) foram associados a um risco reduzido de DAP.

### Influência de 91 citocinas inflamatórias nas características da DRC (Doença Renal Crônica)

**Figura 2:**
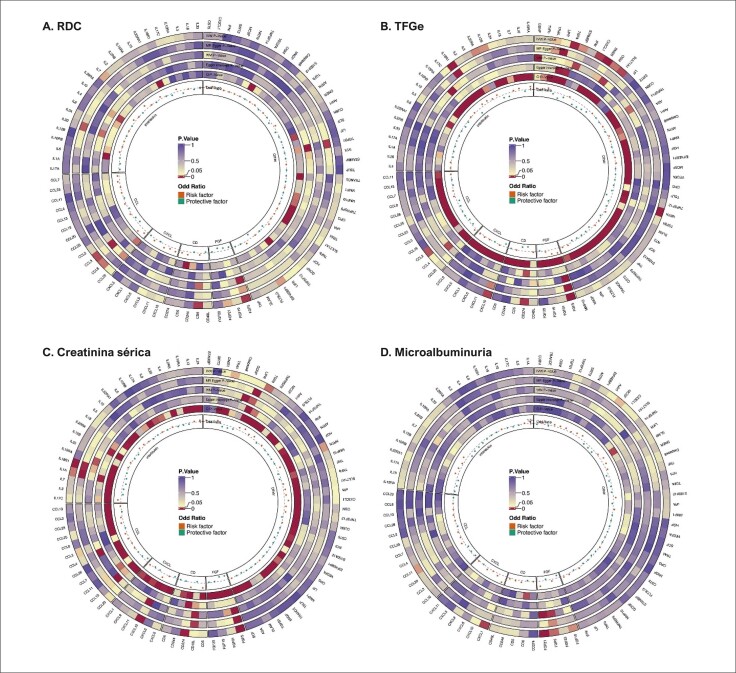
– Resultados do mapa de calor anular das 91 citocinas inflamatórias sobre as características da DRC (Doença Renal Crônica). DRC: doença renal crônica; TFGe: taxa de filtração glomerular estimada.

A Figura 2 apresenta as associações entre citocinas e características da DRC por meio de visualização em mapa de calor anular. Para a DRC (Figura 2A), CCL8 e CD6 emergiram como mediadores de risco, contrastando com os efeitos protetores de CCL3, EIF4EBP1, FGF21, FGF5 e IL7. As análises de TFGe delinearam mecanismos regulatórios opostos (Figura 2B): CCL2, CD274, CX3CL1, CXCL10, DNER, IL12B, NRTN, OSM, STAMBP, SULT1A1 e TGFA diminuíram os níveis de TFGe, enquanto FGF5, IL18R1, IL20RA e TRAIL melhoraram a função renal. As análises de creatinina sérica revelaram que CCL20, FGF5, IL18R1, IL1A e TRAIL reduziram o acúmulo de creatinina, enquanto CCL2, CD274, CXCL10, NRTN e TGFA exacerbaram a retenção (Figura 2C). O risco de microalbuminúria foi atenuado por ADA, FGF5, FGF19 e MCSF, mas amplificado por CCL11, CXCL1 e FGF21 (Figura 2D). As análises de mediana ponderada e de sensibilidade MR-Egger corroboraram esses achados (Figura Suplementar 3), identificando os efeitos renoprotetores pleiotrópicos do FGF5 em todos os desfechos renais.Depois da correção de Bonferroni, DNER (OR=0,995; IC 95%: 0,993–0,998; p=0,001) e NRTN (OR=0,995; IC 95%: 0,992–0,998; p=0,002) diminuíram o nível de eGFR, enquanto FGF5 (OR=1,004; IC 95%: 1,002–1,006; p<0,001) e IL18R1 (OR=1,002; IC 95%: 1,001–1,003; p=0,001) aumentaram o nível de eGFR. CD274 (OR=1,030; IC 95%: 1,010–1,051; p=0,003) e CXCL10 (OR=1,024; IC 95%: 1,009–1,040; p=0,002) aumentaram o nível de screatinina sérica, enquanto FGF5 (OR=0,975; IC 95%: 0,964–0,987; p<0,001) e IL18R1 (OR=0,989; IC 95%: 0,983–0,996; p=0,002) diminuiu o nível de screatinina sérica. FGF21 (OR=1,448; IC 95%: 1,151–1,821; p=0,002) foi associada a um risco aumentado de microalbuminúria.

### Influência de 91 citocinas inflamatórias em características da obesidade

**Figura 3:**
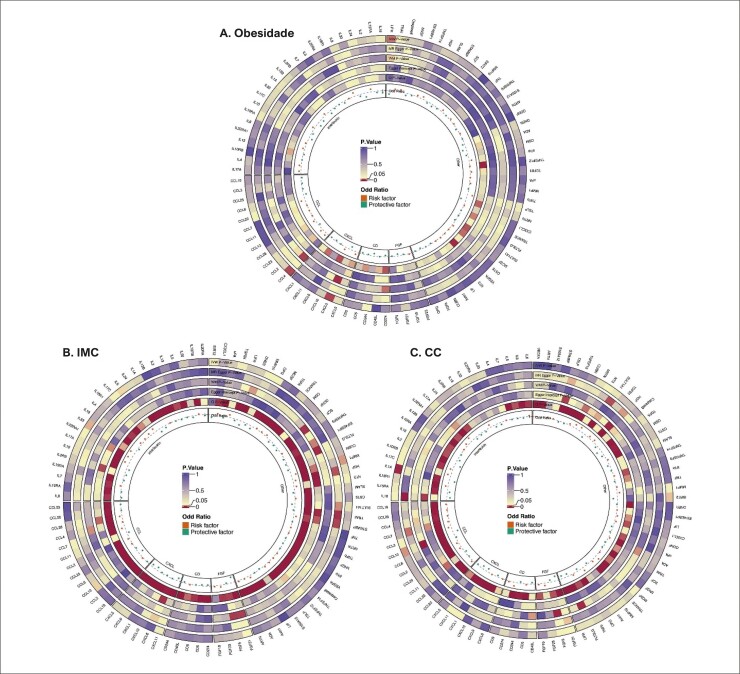
– Resultados do mapa de calor anular das 91 citocinas inflamatórias sobre características da obesidade. IMC: índice de massa corporal; CC: circunferência da cintura.

A Figura 3 ilustra as associações entre citocinas e obesidade por meio de análise de mapa de calor anular. As análises de obesidade identificaram CCL4 como o principal mediador de risco para obesidade, com CXCL5 e LIFR demonstrando efeitos protetores (Figura 3A). As análises de IMC revelaram IL6, IL33 e MMP1 como reguladores positivos, contrastando com o papel limitador da adiposidade de CCL13 (Figura 3B). As análises de CC revelaram que IL17C promoveu adiposidade central, enquanto CCL13, IL1A e SIRT2 conferiram proteção metabólica (Figura 3C). Análises de sensibilidade por meio dos métodos de mediana ponderada e MR-Egger confirmaram a robustez em todas as métricas de obesidade (Figura Suplementar 4), destacando os efeitos protetores duplos de CCL13 tanto no IMC quanto na CC.Depois da correção de Bonferroni: nenhuma associação permaneceu significativa.

### Influência de 91 citocinas inflamatórias nas características do diabetes

**Figura 4:**
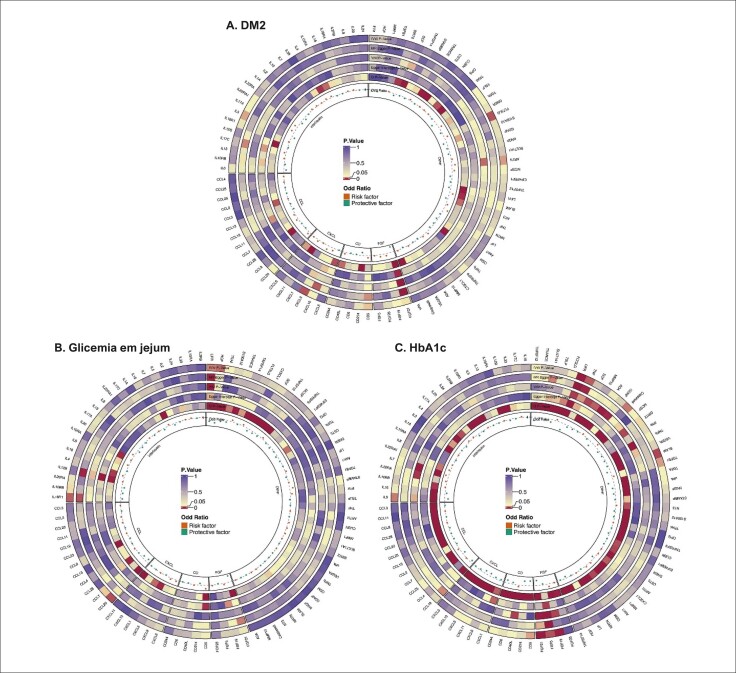
– Resultados do mapa de calor anular das 91 citocinas inflamatórias sobre características do diabetes. Hb A1c: hemoglobina glicada A1c; DM2: diabetes tipo 2.

A Figura 4 descreve as interações entre citocinas e diabetes por meio de análise de mapa de calor anular. As análises de diabetes identificaram ARTN, CD5, CXCL9 e FLT3LG como mediadores patogênicos para o risco de diabetes, em contraste com os efeitos protetores de FGF21 e IL5 (Figura 4A). A regulação da glicemia em jejum envolveu CCL25, CCL7, HGF, IL10RB, IL18R1 e LIFR, demonstrando controle glicêmico (Figura 4B). As análises de HbA1c revelaram regulação bidirecional: FGF19, IL7 e TGFB1 elevaram os níveis de glicação, enquanto Caspase8, CD5, FGF21, FGF5, IL6, LIFR, SIRT2, SLAM e TNF exibiram benefícios glicêmicos (Figura 4C). As análises de mediana ponderada e de sensibilidade MR-Egger validaram essas associações (Figura Suplementar 5), identificando o FGF21 como tendo potencial terapêutico duplo por meio da redução do risco de diabetes e da diminuição da HbA1c.DepoisBonferroniA correção, FGF19 (OR=1,024; IC 95%: 1,008–1,041; p=0,003) foi associada a maiornível de HbA1c, enquanto FGF21(OR=0,951; IC 95%: 0,921–0,981; p=0,001)e LIFR(OR=0,933; IC 95%: 0,898–0,969; p<0,001) foram associados a menornível de HbA1c.

O desenho geral do estudo pode ser encontrado na Figura Central.

## Discussão

Nossa investigação multidimensional por ressonância magnética resultou em quatro avanços cruciais para a compreensão da regulação inflamatória da síndrome de CKM: 1) delineamento sistemático de 60 citocinas associadas à doença, estabelecendo alvos terapêuticos específicos para cada contexto; 2) identificação de FGF5 e IL6 como reguladores paradoxais com efeitos opostos em diferentes sistemas orgânicos; e 3) validação do MCSF como um amplificador de risco pan-vascular. Essas descobertas desafiam dicotomias simplistas pró/anti-inflamatórias, revelando, em vez disso, uma interação complexa de pleiotropia de citocinas, moldada pela biologia de receptores específicos de cada tecido e pela dinâmica temporal.

Nossos resultados exigem uma reconceitualização fundamental da biologia inflamatória, transcendendo o dogma antiquado de “pró-inflamatório = patogênico”, ao demonstrar que as citocinas operam como biossensores sensíveis ao contexto, cujas respostas funcionais são ditadas pela topografia do receptor específico do tecido e pela dinâmica de exposição temporal. O paradoxo do FGF5 exemplifica esse paradigma, onde FGF5 foi identificado como um fator de risco para doenças cardiovasculares, incluindo FA e doença coronariana, um fator protetor para doença renal crônica (DRC, TFGe, creatinina sérica e microalbuminúria) e para o controle da HbA1c.A proteína FGF5 é um membro da família do fator de crescimento de fibroblastos que estimula o crescimento e a proliferação celular em múltiplos tipos de células, incluindo miócitos cardíacos, e tem sido associada à hipertrofia e angiogênese no miocárdio hibernante.^29^Essa aparente contradição em nosso estudo reflete evidências recentes de RM que mostram a modulação tecido-específica do FGF5. Análises de RM e de colocalização indicaram que níveis geneticamente determinados de FGF5 estavam associados a um risco aumentado de DAC (OR=1,08; IC 95%: 1,05-1,11).^30^ Da mesma forma, o SNP cis-pQTL usado como instrumento genético para FGF5 também foi associado à DAC.^31^ No entanto, utilizando pQTLs de 3032 proteínas provenientes de 3 grandes estudos de GWAS e os respectivos eQTLs específicos para sangue e tecido, a expressão gênica de FGF5 apresentou associação negativa com a DRC em amostras do córtex renal.^32,33^ Enquanto isso, uma maior concentração de FGF5 geneticamente prevista foi associada a uma TFGe mais elevada.^33^ Esses achados alertam contra a modulação sistêmica da via do FGF, defendendo, em vez disso, sistemas de administração direcionados a tecidos, atualmente em desenvolvimento.^34^ A associação protetora da IL-6 com a DAC parece inicialmente discordante com estudos observacionais que relacionam a IL-6 elevada ao risco cardiovascular.^35^ Embora picos agudos de IL-6 promovam a instabilidade da placa, a elevação moderada ao longo da vida (como modelada pela RM) pode induzir a remodelação vascular adaptativa. A hormese, definida como uma resposta adaptativa de células e organismos a um estresse moderado, pode ativar mecanismos cardioprotetores.^36^ Nossos achados também são consistentes com o crescente reconhecimento dos efeitos diferenciais das espécies reativas de oxigênio “boas” e “ruins” nos benefícios para a saúde.^37^

Nosso estudo destaca o MCSF (codificado por CSF1) como um amplificador crucial e consistente do risco vascular em importantes doenças ateroscleróticas, incluindo doença coronariana, AVC e DAP. Em consonância com nosso estudo, o valor prognóstico do MCSF foi independente e complementar ao da proteína C-reativa (PCR) em 100 pacientes com doença coronariana crônica.^38^ Macrófagos ativados por MCSF migram para a capa fibrosa de uma placa aterosclerótica, produzem metaloproteinases e, assim, podem causar a desestabilização da placa.^39^ O MCSF também promove a ativação plaquetária,^40^ a expressão do fator tecidual^41^ e a liberação da citocina pró-coagulante.^42^ Por meio dessas ações, o MCSF pode promover a trombose e, assim, contribuir para o desenvolvimento de síndromes coronárias agudas e AVC isquêmico em pacientes com aterosclerose. O direcionamento do MCSF pode representar uma nova estratégia terapêutica para reduzir o risco vascular global. Estudos pré-clínicos demonstraram que a deleção genética ou a inibição farmacológica do MCSF atenua a aterosclerose em modelos animais, reduzindo a carga de placa e a inflamação.^43-45^ A transposição desses achados para a prática clínica requer cautela e validação adicional.

Para fornecer informações mecanísticas, as citocinas atuam por meio de vias de sinalização específicas de tecido e contexto que moldam a progressão da CKM. A IL-6 sinaliza por meio de modos clássico e trans, com a sinalização clássica mediando efeitos homeostáticos, enquanto a sinalização trans promove a ativação endotelial, o recrutamento de monócitos e a inflamação crônica, ativando as vias JAK/STAT3, PI3K/AKT e MAPK.^46,47^ O FGF5 modula a proliferação celular e a remodelação vascular de maneira tecido-dependente, explicando seus efeitos pleiotrópicos. O MCSF impulsiona o recrutamento e a ativação de macrófagos, além da desestabilização da placa, contribuindo para o risco vascular.^48^ Essas descobertas destacam que as citocinas funcionam como reguladores dinâmicos e sensíveis ao contexto, onde a exposição temporal e a expressão de receptores tecido-específicos determinam papéis patogênicos versus protetores, corroborando a classificação da CKM como uma síndrome sistêmica, e não como uma doença isolada de um órgão.

Embora nossos resultados resistam a análises de sensibilidade rigorosas, algumas considerações metodológicas merecem ser reconhecidas. Primeiro, as relações dose-resposta não lineares das citocinas permanecem não caracterizadas devido às limitações inerentes às abordagens de RM em nível de resumo, o que impede a avaliação de efeitos de limiar ou associações em forma de U. Segundo, nossas estimativas refletem a predisposição genética ao longo da vida para a modulação de citocinas, que pode não espelhar diretamente os efeitos terapêuticos da modulação farmacológica aguda de citocinas. Terceiro, os efeitos dimórficos sexuais não puderam ser determinados devido às fontes de GWAS predominantemente mistas em relação aos sexos. Quarto, os instrumentos genéticos para citocinas inflamatórias foram derivados exclusivamente de coortes de ascendência europeia, limitando, portanto, a generalização de nossos resultados para outros grupos ancestrais.^11^ Quinto, cálculos formais de poder estatístico da RM não foram realizados, o que pode limitar a capacidade de detectar associações modestas. Estudos futuros com amostras maiores são necessários para validar esses resultados exploratórios. Finalmente, dado o grande número de testes realizados, alguns resultados podem estar sujeitos a viés de falso-positivo. Embora tenha sido realizada uma correção de Bonferroni, o número reduzido de associações significativas após o ajuste destaca a necessidade de uma interpretação cautelosa e de replicação independente.

## Conclusões

Este atlas de RM redefine as redes inflamatórias em doenças cardiovasculares, renais e metabólicas, revelando o FGF5 e a IL6 como reguladores paradoxais que requerem modulação específica ao contexto, e o MCSF como um amplificador unificador do risco vascular. Nossos resultados desafiam as dicotomias simplistas pró/anti-inflamatórias, revelando, em vez disso, uma interação complexa de pleiotropia de citocinas moldada pela biologia de receptores específicos de tecido e pela dinâmica temporal na era CKM.

## Material suplementar

Supplementary Figure 1. The overview of the study design.
