# Supplementary material for: Investigando o Papel Causal das Citocinas Inflamatórias no Desenvolvimento de Doenças Cardiovasculares, Renais e Metabólicas
Source: Arq Bras Cardiol. 2026 May 26;123(4):e20250611. [Article in Portuguese] doi: 10.36660/abc.20250611 (PMC13398828; doi:10.36660/abc.20250611)
Supplement: Supplementary Figure 1. The overview of the study design. [file 0066-782x-abc-123-4-e20250611-suppl01.pdf]

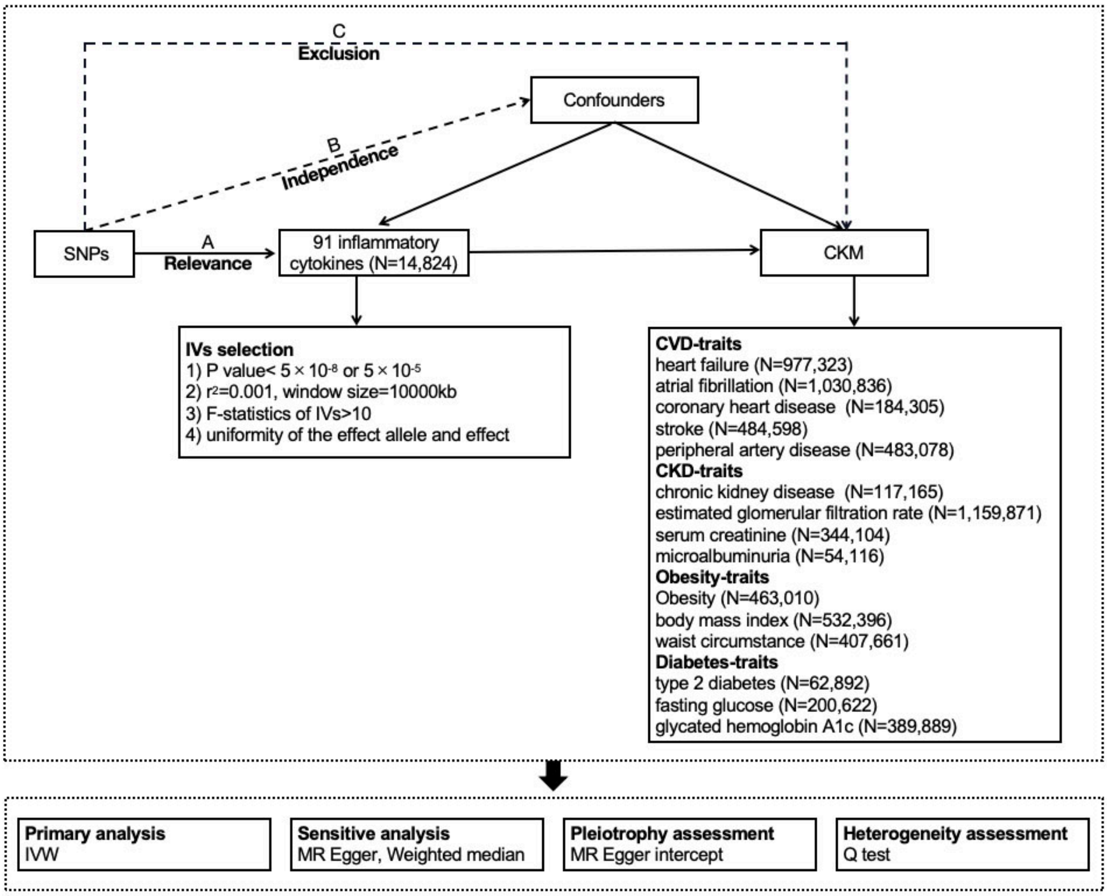

Supplementary Figure 1. The overview of the study design.

CKD: chronic kidney disease; CKM: cardiovascular-kidney-metabolic syndrome; CVD: cardiovascular disease; IV: instrumental variable; IVW: inverse variance weighting; MR: Mendelian randomization; SNP: single nucleotide polymorphisms

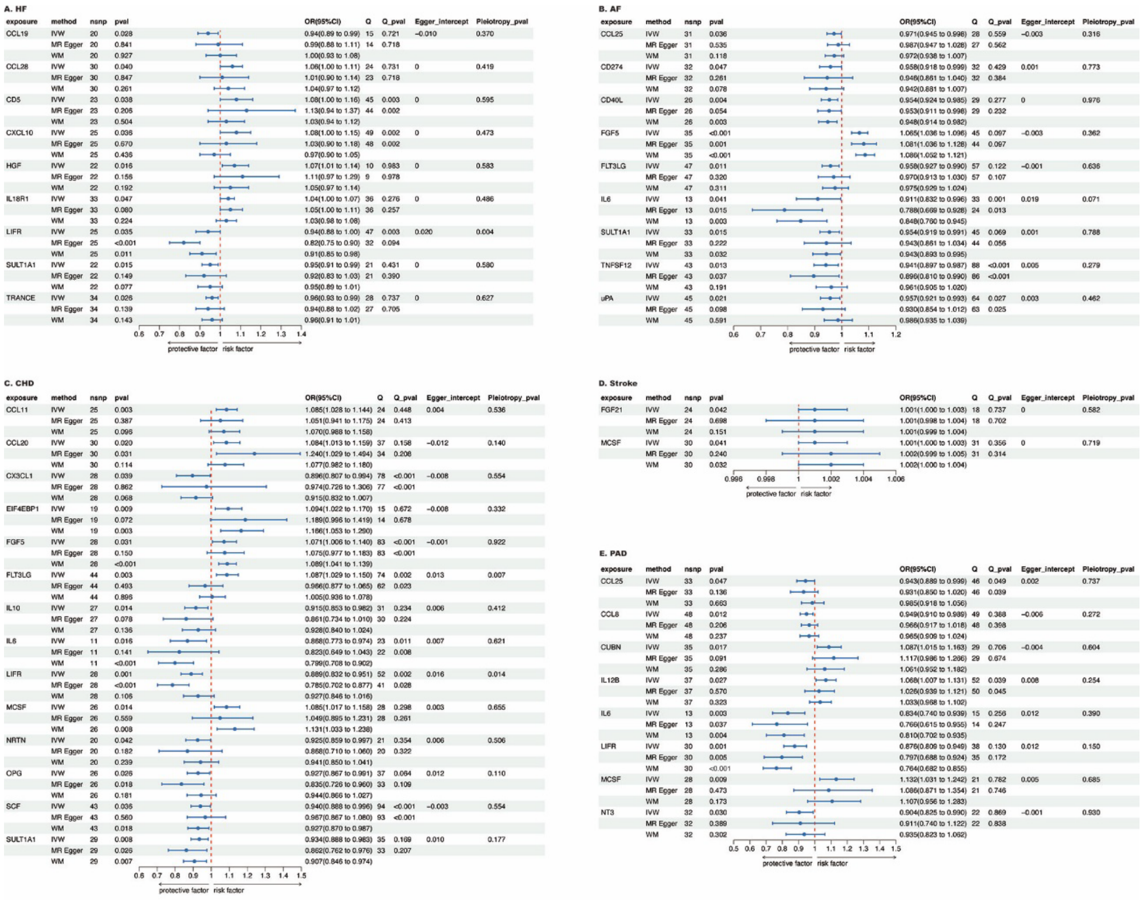

Supplementary Figure 2. Sensitivity analysis of the effect of 91 inflammatory cytokines on CVD traits

AF: atrial fibrillation; CHD: coronary heart disease; CVD: cardiovascular disease; HF: heart failure; PAD: peripheral artery disease; WM: weighted median

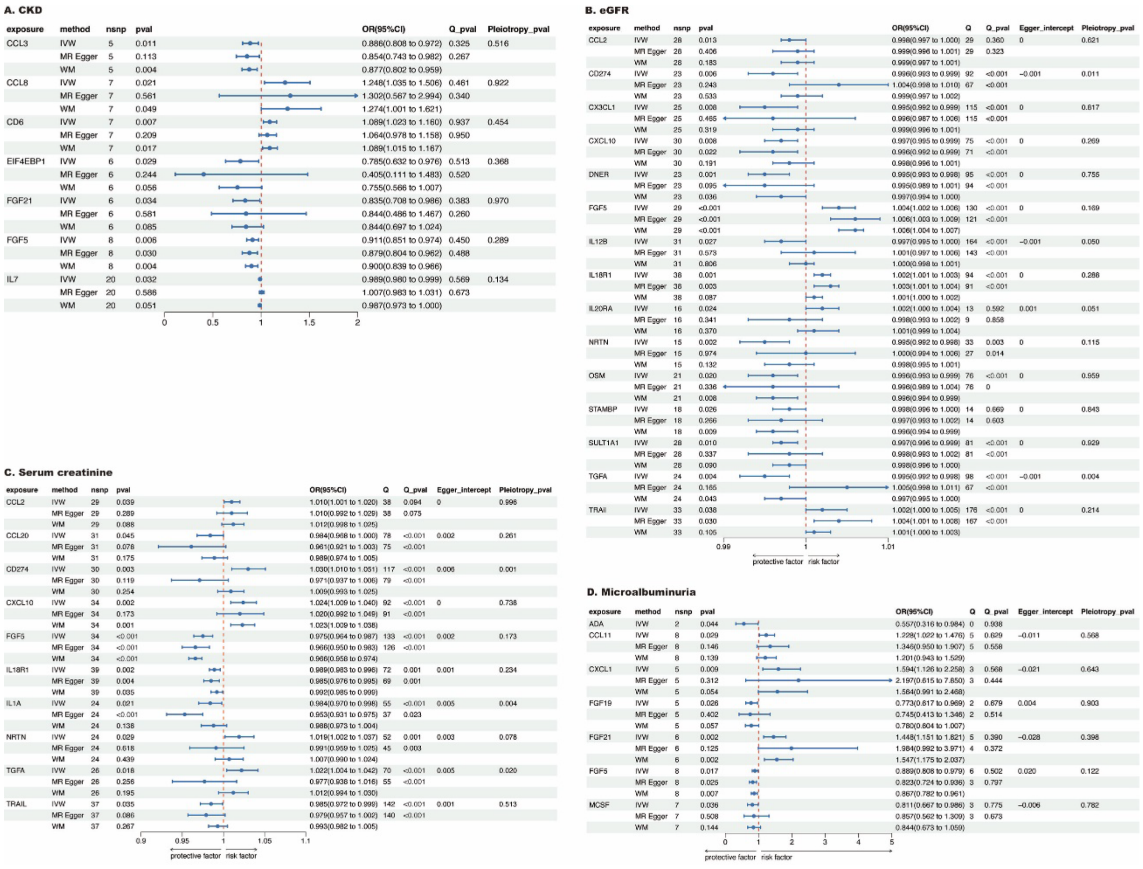

Supplementary Figure 3. Sensitivity analysis of the effect of 91 inflammatory cytokines on CKD traits

CKD: chronic kidney disease; eGFR: estimated glomerular filtration rate; WM: weighted median

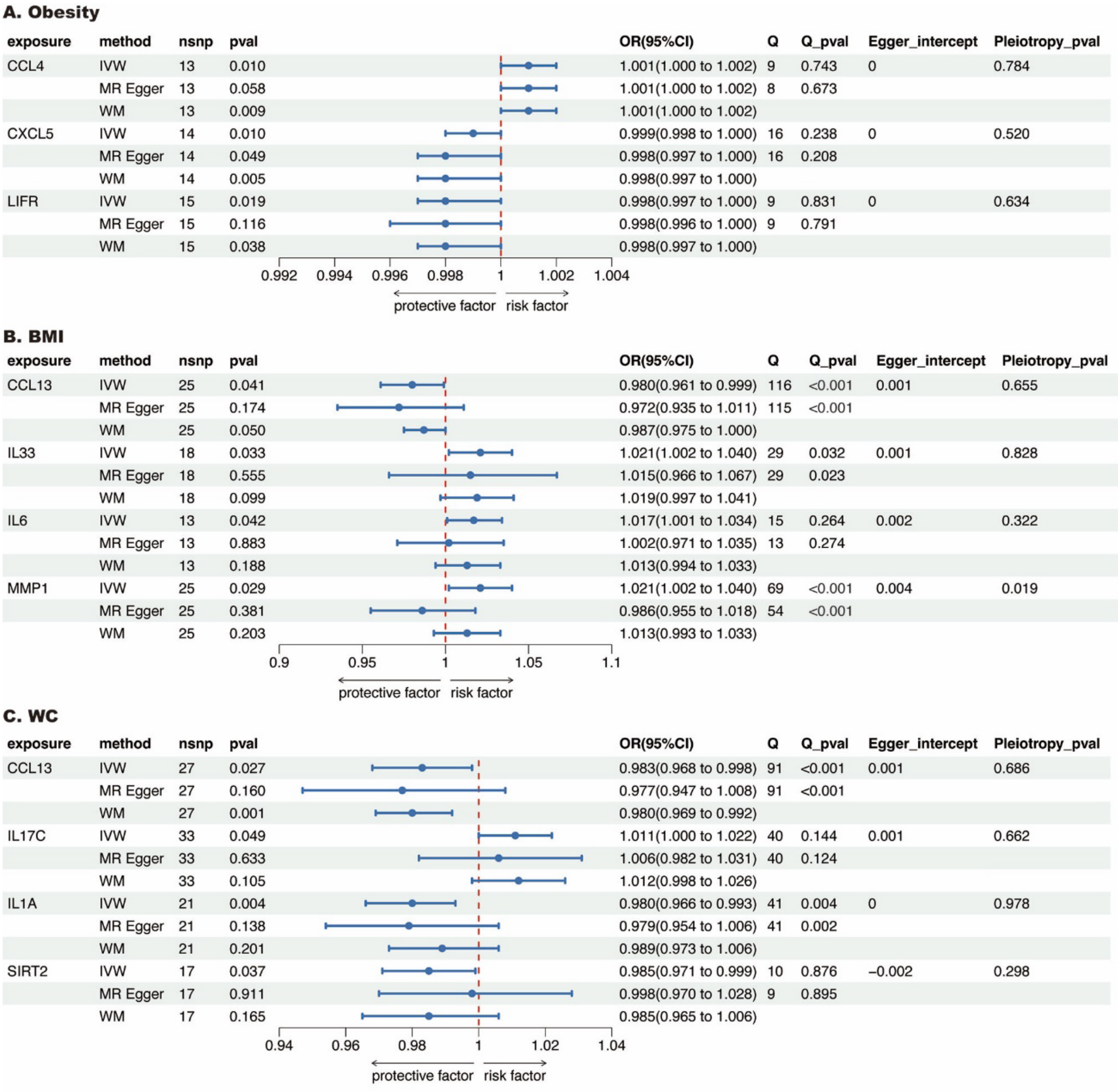

A. Obesity

exposure	method	nsnp	pval	OR(95%CI)	Q	Q_pval	Egger_intercept	Pleiotropy_pval
CCL4	IVW	13	0.010	1.001(1.000 to 1.002)	9	0.743	0	0.784
	MR Egger	13	0.058	1.001(1.000 to 1.002)	8	0.673		
	WM	13	0.009	1.001(1.000 to 1.002)				
CXCL5	IVW	14	0.010	0.999(0.998 to 1.000)	16	0.238	0	0.520
	MR Egger	14	0.049	0.998(0.997 to 1.000)	16	0.208		
	WM	14	0.005	0.998(0.997 to 1.000)				
LIFR	IVW	15	0.019	0.998(0.997 to 1.000)	9	0.831	0	0.634
	MR Egger	15	0.116	0.998(0.996 to 1.000)	9	0.791		
	WM	15	0.038	0.998(0.997 to 1.000)				

B. BMI

exposure	method	nsnp	pval	OR(95%CI)	Q	Q_pval	Egger_intercept	Pleiotropy_pval
CCL13	IVW	25	0.041	0.980(0.961 to 0.999)	116	<0.001	0.001	0.655
	MR Egger	25	0.174	0.972(0.935 to 1.011)	115	<0.001		
	WM	25	0.050	0.987(0.975 to 1.000)				
IL33	IVW	18	0.033	1.021(1.002 to 1.040)	29	0.032	0.001	0.828
	MR Egger	18	0.555	1.015(0.966 to 1.067)	29	0.023		
	WM	18	0.099	1.019(0.997 to 1.041)				
IL6	IVW	13	0.042	1.017(1.001 to 1.034)	15	0.264	0.002	0.322
	MR Egger	13	0.883	1.002(0.971 to 1.035)	13	0.274		
	WM	13	0.188	1.013(0.994 to 1.033)				
MMP1	IVW	25	0.029	1.021(1.002 to 1.040)	69	<0.001	0.004	0.019
	MR Egger	25	0.381	0.986(0.955 to 1.018)	54	<0.001		
	WM	25	0.203	1.013(0.993 to 1.033)				

C. WC

exposure	method	nsnp	pval	OR(95%CI)	Q	Q_pval	Egger_intercept	Pleiotropy_pval
CCL13	IVW	27	0.027	0.983(0.968 to 0.998)	91	<0.001	0.001	0.686
	MR Egger	27	0.160	0.977(0.947 to 1.008)	91	<0.001		
	WM	27	0.001	0.980(0.969 to 0.992)				
IL17C	IVW	33	0.049	1.011(1.000 to 1.022)	40	0.144	0.001	0.662
	MR Egger	33	0.633	1.006(0.982 to 1.031)	40	0.124		
	WM	33	0.105	1.012(0.998 to 1.026)				
IL1A	IVW	21	0.004	0.980(0.966 to 0.993)	41	0.004	0	0.978
	MR Egger	21	0.138	0.979(0.954 to 1.006)	41	0.002		
	WM	21	0.201	0.989(0.973 to 1.006)				
SIRT2	IVW	17	0.037	0.985(0.971 to 0.999)	10	0.876	−0.002	0.298
	MR Egger	17	0.911	0.998(0.970 to 1.028)	9	0.895		
	WM	17	0.165	0.985(0.965 to 1.006)				

Supplementary Figure 4. Sensitivity analysis of the effect of 91 inflammatory cytokines on obesity traits

BMI: body mass index; WC: waist circumstance; WM: weighted median

A. T2D

exposure	method	nsnp	pval	OR(95%CI)	Q	Q_pval	Egger_intercept	Pleiotropy_pval
ARTN	IVW	13	0.029	1.093(1.009 to 1.184)	16	0.189	−0.016	0.454
	MR Egger	13	0.294	1.339(0.796 to 2.251)	15	0.173		
	WM	13	0.017	1.128(1.022 to 1.246)				
CD5	IVW	12	0.038	1.069(1.004 to 1.139)	13	0.324	−0.014	0.083
	MR Egger	12	0.026	1.237(1.054 to 1.450)	9	0.548		
	WM	12	0.059	1.089(0.997 to 1.189)				
CXCL9	IVW	11	0.012	1.113(1.023 to 1.211)	16	0.109	−0.007	0.459
	MR Egger	11	0.138	1.211(0.962 to 1.525)	15	0.099		
	WM	11	0.039	1.112(1.005 to 1.230)				
FGF21	IVW	11	0.045	0.834(0.699 to 0.996)	80	<0.001	0.050	0.004
	MR Egger	11	0.001	0.494(0.368 to 0.663)	31	<0.001		
	WM	11	0.001	0.820(0.731 to 0.921)				
FLT3LG	IVW	17	0.013	1.097(1.020 to 1.180)	23	0.102	0.012	0.259
	MR Egger	17	0.660	0.941(0.721 to 1.228)	21	0.122		
	WM	17	0.066	1.088(0.994 to 1.190)				
IL5	IVW	9	0.031	0.913(0.841 to 0.992)	7	0.556	0.005	0.749
	MR Egger	9	0.432	0.863(0.609 to 1.222)	7	0.460		
	WM	9	0.169	0.926(0.829 to 1.033)				

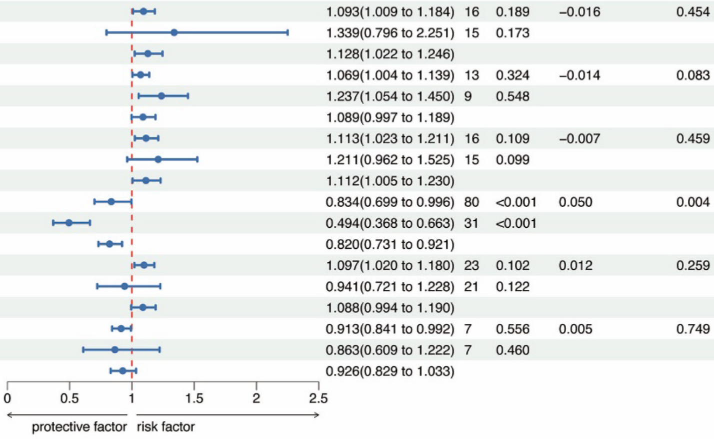

B. Fasting glucose

exposure	method	nsnp	pval	OR(95%CI)	Q	Q_pval	Egger_intercept	Pleiotropy_pval
CCL25	IVW	34	0.011	0.985(0.974 to 0.997)	91	<0.001	0.001	0.562
	MR Egger	34	0.045	0.981(0.964 to 0.999)	90	<0.001		
	WM	34	0.206	0.994(0.984 to 1.004)				
CCL7	IVW	31	0.039	0.988(0.977 to 0.999)	35	0.232	0	0.768
	MR Egger	31	0.555	0.992(0.966 to 1.019)	35	0.198		
	WM	31	0.099	0.986(0.971 to 1.003)				
HGF	IVW	35	0.018	0.977(0.958 to 0.996)	81	<0.001	0.001	0.649
	MR Egger	35	0.156	0.968(0.926 to 1.011)	80	<0.001		
	WM	35	0.135	0.986(0.968 to 1.004)				
IL10RB	IVW	28	0.043	0.992(0.984 to 1.000)	34	0.174	−0.001	0.381
	MR Egger	28	0.430	0.995(0.984 to 1.007)	33	0.17		
	WM	28	0.142	0.993(0.985 to 1.002)				
IL18R1	IVW	39	0.015	0.992(0.986 to 0.999)	41	0.337	0.001	0.209
	MR Egger	39	0.012	0.988(0.980 to 0.997)	39	0.365		
	WM	39	0.058	0.993(0.985 to 1.000)				
LIFR	IVW	30	0.024	0.982(0.967 to 0.998)	89	<0.001	0.003	0.034
	MR Egger	30	0.003	0.958(0.933 to 0.983)	76	<0.001		
	WM	30	0.002	0.971(0.952 to 0.989)				

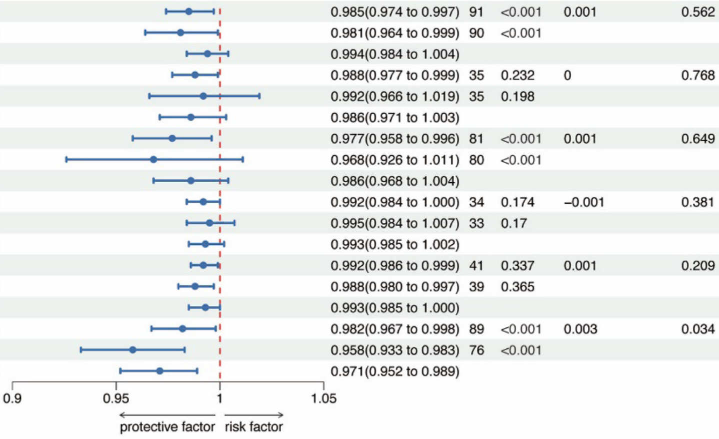

C. HbA1c

exposure	method	nsnp	pval	OR(95%CI)	Q	Q_pval	Egger_intercept	Pleiotropy_pval
CD5	IVW	27	0.030	0.953(0.912 to 0.995)	364	<0.001	0.003	0.513
	MR Egger	27	0.146	0.923(0.831 to 1.025)	357	<0.001		
	WM	27	0.997	1.000(0.980 to 1.020)				
Caspase8	IVW	16	0.021	0.958(0.923 to 0.994)	65	<0.001	−0.005	0.171
	MR Egger	16	0.741	1.015(0.931 to 1.107)	57	<0.001		
	WM	16	0.068	0.976(0.951 to 1.002)				
FGF19	IVW	32	0.003	1.024(1.008 to 1.041)	69	<0.001	0.002	0.337
	MR Egger	32	0.705	1.007(0.970 to 1.046)	67	<0.001		
	WM	32	0.009	1.025(1.006 to 1.043)				
FGF21	IVW	25	0.001	0.951(0.921 to 0.981)	188	<0.001	0.003	0.377
	MR Egger	25	0.034	0.925(0.864 to 0.990)	182	<0.001		
	WM	25	0.003	0.968(0.948 to 0.989)				
FGF5	IVW	29	0.012	0.990(0.982 to 0.998)	32	0.272	0.003	0.008
	MR Egger	29	0.001	0.978(0.967 to 0.989)	24	0.638		
	WM	29	<0.001	0.983(0.974 to 0.992)				
IL6	IVW	13	0.022	0.968(0.941 to 0.995)	40	<0.001	0.002	0.676
	MR Egger	13	0.164	0.957(0.904 to 1.014)	39	<0.001		
	WM	13	0.033	0.968(0.939 to 0.997)				
IL7	IVW	24	0.049	1.026(1.000 to 1.053)	85	<0.001	0.004	0.168
	MR Egger	24	0.584	0.981(0.918 to 1.049)	78	<0.001		
	WM	24	0.002	1.034(1.012 to 1.057)				
LIFR	IVW	27	<0.001	0.933(0.898 to 0.969)	345	<0.001	0.011	0.001
	MR Egger	27	<0.001	0.847(0.799 to 0.899)	220	<0.001		
	WM	27	0.007	0.970(0.949 to 0.992)				
SIRT2	IVW	17	0.043	0.983(0.968 to 0.999)	17	0.379	0	0.785
	MR Egger	17	0.239	0.979(0.947 to 1.013)	17	0.318		
	WM	17	0.010	0.970(0.948 to 0.993)				
SLAM	IVW	37	0.010	0.968(0.944 to 0.992)	243	<0.001	−0.001	0.826
	MR Egger	37	0.312	0.973(0.923 to 1.026)	243	<0.001		
	WM	37	0.263	0.991(0.974 to 1.007)				
TGFB1	IVW	29	0.029	1.016(1.002 to 1.030)	40	0.062	0	0.864
	MR Egger	29	0.309	1.014(0.988 to 1.041)	40	0.048		
	WM	29	0.132	1.015(0.995 to 1.035)				
TNF	IVW	24	0.014	0.983(0.969 to 0.997)	29	0.192	0.003	0.033
	MR Egger	24	0.004	0.954(0.927 to 0.982)	23	0.390		
	WM	24	0.027	0.979(0.961 to 0.998)				

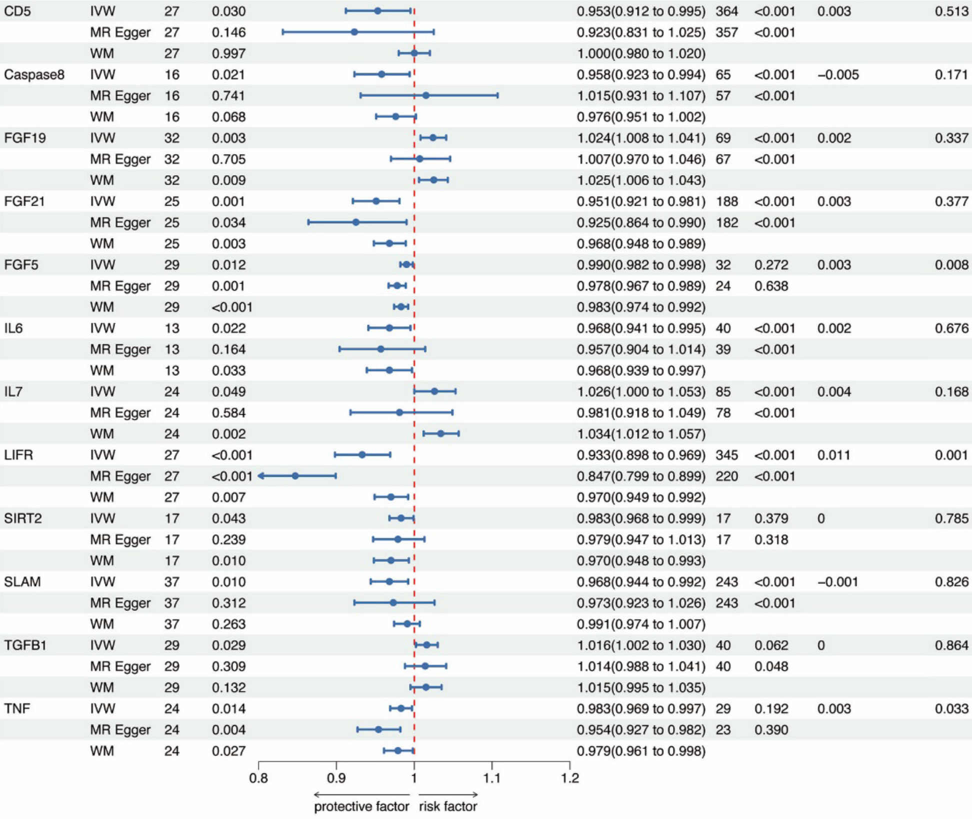

Supplementary Figure 5. Sensitivity analysis of the effect of 91 inflammatory cytokines on diabetes traits

Hb A1c: glycated hemoglobin A1c; T2D: type 2 diabetes; WM: weighted median
