# Supplementary material for: Investigando o Papel Causal das Citocinas Inflamatórias no Desenvolvimento de Doenças Cardiovasculares, Renais e Metabólicas
Source: Arq Bras Cardiol. 2026 May 26;123(4):e20250611. [Article in Portuguese] doi: 10.36660/abc.20250611 (PMC13398828; doi:10.36660/abc.20250611)
Supplement: Translation

# Translation: Investigating the Causal Role of Inflammatory Cytokines in the Development of Cardiovascular, Kidney, and Metabolic Diseases

**DOI:** 10.36660/abc.20250611i

**Authors:** Mengjin Hu, Zhaoting Gong, Xiaosong Li, Chunlin Yin

**Affiliations:** 1 China Xuanwu Hospital, Capital Medical University, Beijing – China; 2 China Beijing Anzhen Hospital, Capital Medical University, Beijing – China

**Keywords:** Cytokines, Inflammation, Cardiovascular Diseases, Chronic Renal Insufficiency, Mendelian Randomization Analysis, Interleukin-6

## Abstract

**Background:**

Observational studies have linked inflammatory cytokines to cardiometabolic disorders; however, whether these associations reflect causal effects across interconnected organ systems remains unclear.

**Objectives:**

This Mendelian randomization (MR) study investigates causality between 91 inflammatory cytokines and multiorgan dysfunction across cardiovascular, kidney, and metabolic systems in the cardiovascular-kidney-metabolic (CKM) syndrome era.

**Methods:**

Genetic instruments associated with 91 inflammatory cytokines were obtained from the Olink Target Inflammation panel across 11 European-ancestry cohorts. The inverse variance weighting (IVW) method was considered as the main analysis, complemented by sensitivity analyses including MR-Egger and weighted median methods.

**Results:**

Genetically predicted levels of inflammatory cytokines showed widespread causal associations across CKM-related traits. Specifically, 31 cytokines were associated with cardiovascular outcomes, 26 with kidney traits, 10 with obesity-related traits, and 21 with diabetes-related traits, yielding a total of 60 cytokines with at least one significant association. Notably, FGF5 and IL6 emerged as paradoxical regulators, exerting opposing effects across different organ systems, while MCSF was consistently identified as a pan-vascular risk amplifier for cardiovascular diseases.

**Conclusions:**

This comprehensive MR analysis reveals substantial causal heterogeneity in inflammatory cytokine effects across cardiovascular, kidney, and metabolic systems. These findings highlight the clinical importance of tissue- and context-specific inflammatory modulation, supporting the development of precision therapeutic strategies for CKM syndrome.

## Introduction

Obesity, diabetes, and chronic kidney disease (CKD) constitute a triad of epidemiologically interconnected conditions that collectively amplify cardiovascular disease (CVD) morbidity and mortality through synergistic pathophysiology.^1^ In recognition of these complex interactions, the American Heart Association has recently established cardiovascular-kidney-metabolic (CKM) syndrome as a novel diagnostic entity – a multisystem disorder characterized by the dynamic interplay of metabolic dysregulation, cardiovascular compromise, and renal dysfunction.^2^ Importantly, CKM syndrome is not defined as a single disease entity but rather as a clinical continuum encompassing obesity, diabetes mellitus, CKD, and their downstream cardiovascular sequelae, including heart failure (HF), atrial fibrillation (AF), coronary heart disease (CHD), stroke, and peripheral artery disease (PAD).^2^ This conceptualization underscores the shared biological pathways and progressive nature of cardiometabolic and renal dysfunction across organ systems. Consistent with its broad clinical relevance, analyses of National Health and Nutrition Examination Survey (NHANES) 2011-2018 data indicate that CKM risk factors or subclinical manifestations (stages 1–3) are present in 80.94% of young adults (20-44 years), 85.95% of middle-aged individuals (45-64 years), and 72.03% of elderly populations (≥65 years).^3^

From a research perspective, however, CKM syndrome currently lacks a unified and validated genetic phenotype suitable for causal inference analyses. Consequently, we attempted to operationalize CKM by examining representative cardiovascular, kidney, and metabolic traits individually, while interpreting them within an integrated multisystem framework. This trait-based approach enables systematic evaluation of shared and divergent mechanisms across organ systems and aligns with the contemporary understanding of CKM as a systems-level continuum rather than a singular nosological entity.

Elucidating the pathophysiological mechanisms underlying these interconnected disorders is crucial for developing targeted therapeutic strategies and improving patient outcomes. Recent research has increasingly focused on the underlying inflammatory mechanisms that link these diseases.^4,5^ Nevertheless, establishing definitive causal relationships remains challenging in observational research due to persistent methodological constraints, including unanticipated confounding variables and reverse causation.

Recent methodological advancements have established Mendelian randomization (MR) as a robust analytical paradigm for causal inference in epidemiological research.^6-8^ This approach utilizes the quasi-random segregation of genetic variants during gametogenesis as nature’s randomization mechanism, enabling causal estimations that approximate randomized controlled trials. By using genetic variants as instrumental variables, MR^9^ can effectively mitigate confounding bias and reverse causation that often plagues observational studies.^10^ MR has been extensively utilized to investigate CVD risk factors and to assess the causal effects of therapeutic interventions.^9,11^ In this study, we first extracted valid genetic variants from the published genome-wide association study (GWAS) summary data of 91 inflammatory cytokines to investigate their correlations with cardiovascular, kidney, and metabolic diseases.

## Methods

Our MR investigation adhered to the STROBE-MR reporting guidelines,^12^ fulfilling three fundamental epidemiological assumptions required for valid causal inference: A) instrument relevance: genetic variants demonstrate genome-wide significant associations with exposure; B) confounder independence: genetic variants are not related to any confounders in the risk factor–outcome association; C) exclusion restriction: genetic variants are not connected with the outcome via any pathways other than the risk factor of interest. The overview of the study design is shown in Supplementary Figure 1.

### Data sources

GWAS data of 91 inflammatory cytokines were quantified using the Olink Target Inflammation panel across 11 European-ancestry cohorts (N=14,824).^13^ CVD analyses incorporated HF (47,309 cases and 930,014 controls),^14^ AF (60,620 cases and 970,216 controls),^15^ CHD (CARDIoGRAMplusC4D Consortium, 60,801 cases and 123,504 controls),^16^ stroke (UK Biobank, 6,925 cases and 477,673 controls),^17^ and PAD (7,114 cases and 475,964 controls).^18^ CKD traits included CKD (FinnGen cohort, 3,902 cases and 212,841 controls), estimated glomerular filtration rate (eGFR, CKDGen Consortium and UK Biobank, 1,159,871 participants),^19^ serum creatinine (FinnGen and UK Biobank, 344,104 participants),^18^ microalbuminuria (CKDGen Consortium, 54,116 participants).^20^ Obesity traits included obesity (UK Biobank, 4,688 cases and 458,322 controls), body mass index (BMI, 532,396 participants),^21^ waist circumference (WC, UK Biobank, 407,661 participants).^22^ Diabetes traits included type 2 diabetes (T2D, 61,714 cases and 1,178 controls),^23^ fasting glucose (200,622 participants),^24^ and glycated hemoglobin A1c (Hb A1c, UK Biobank, 389,889 participants).^22^ All analyses utilized publicly available summary statistics with prior ethical approval and participant consent obtained in original studies through institutional review boards, exempting this study from additional ethical review requirements.

### Instrumental variable selection

Initially, genome-wide significance thresholding (P<5×10^-8^) was employed to identify strongly associated single-nucleotide polymorphisms (SNPs) with inflammatory cytokines. Given limited instrument availability at this stringent threshold, we implemented a liberalized significance criterion (P<1×10^-5^) while maintaining rigorous quality control.^25^ To ensure genetic independence, clumping procedures were performed with parameters set at 10,000 kb window size and r^2^<0.001. Instrument strength was quantified through F-statistic calculations, with variants demonstrating F>10 retained to mitigate weak instrument bias. The F-statistic was calculated using the standard formula $F=(\beta /\mathrm{se}{)}^{2}$ for each genetic instrument. Finally, palindromic SNPs were systematically excluded to maintain consistent allele orientation across exposure-outcome datasets, thereby preventing strand misalignment artifacts.

### Statistical analysis

The inverse variance-weighted (IVW), MR-Egger, and weighted median methods were used to infer causality. The IVW method, which assumes that all genetic variants are valid instrumental variables, was considered the main analysis. In the absence of horizontal pleiotropy, IVW can provide an unbiased causal estimate.^26^ The MR-Egger method can detect and adjust for directional pleiotropy under the INSIDE assumption, but often with reduced statistical power.^27^ The MR-Egger regression was used to assess horizontal pleiotropy. The non-zero intercept of the MR-Egger regression suggested directional pleiotropy.^27^ The weighted median method has a greater tolerance for invalid instrumental variables and can still produce reliable estimates as long as more than half of the total weight comes from valid instrumental variable.^28^ Cochran’s Q test was used to assess the heterogeneity among the instrumental variables. Two-sided p< 0.05 was considered statistically significant for primary analyses. In addition, a Bonferroni-corrected threshold of p < 0.003 (α = 0.05/15 outcomes) was applied to account for multiple comparisons. All MR analyses were performed in R (version 4.4.1) software, using the “TwoSampleMR” package (version 0.6.8).

## Results

### Influence of 91 inflammatory cytokines on CVD-traits

Figure 1 delineates the annular heatmap profiling 91 cytokines across CVD, with IVW analyses revealing distinct risk architectures. Genetically elevated levels of CCL28, CD5, CXCL10, HGF, and IL18R1 (one standard deviation increase) were associated with increased HF risk (Figure 1A), whereas CCL19, LIFR, SULT1A1, and TRANCE demonstrated protective effects. For AF (Figure 1B), FGF5 exhibited risk amplification, contrasting with protective associations for CCL25, CD274, CD40L, FLT3LG, IL6, SULT1A1, TNFSF12, and uPA. CHD analyses identified CX3CL1, IL10, IL6, LIFR, NRTN, OPG, SCF, and SULT1A1 as protective mediators, while CCL11, CCL20, EIF4EBP1, FGF5, FLT3LG, and MCSF emerged as potent atherogenic drivers (Figure 1C). Stroke risk was amplified by FGF21 and MCSF (Figure 1D), whereas PAD exhibited divergent regulation with IL6 conferring protection despite MCSF’s persistent risk effects (Figure 1E). Sensitivity analyses across the MR-Egger and weighted median methods validated these associations (Supplementary Figure 2), highlighting MCSF as a pan-vascular risk amplifier for CVD. Among the numerous significant associations identified, several remained significant after Bonferroni correction. FGF5 (odds ratio [OR]=1.065, 95% confidence interval [CI]: 1.036–1.096; p<0.001) was associated with an increased risk of AF, CCL11 (OR=1.085; 95% CI: 1.028–1.144; p=0.003) and FLT3LG (OR=1.087; 95% CI: 1.029–1.150; p=0.003) were associated with an increased risk of CHD, while LIFR (OR=0.889; 95% CI: 0.832–0.951; P=0.001) was associated with a decreased risk of CDH. IL6 (OR=0.834; 95% CI: 0.740–0.939; p=0.003) and LIFR (OR=0.876; 95% CI: 0.809–0.949; p=0.001) were associated with a decreased risk of PAD.

**Figure 1:** – The annular heat map results of the 91 inflammatory cytokines on CVD traits. AF: atrial fibrillation; CHD: coronary heart disease; CVD: cardiovascular disease; HF: heart failure; PAD: peripheral artery disease.

### Influence of 91 inflammatory cytokines on CKD-traits

Figure 2 presents cytokine-CKD trait associations through annular heatmap visualization. For CKD (Figure 2A), CCL8 and CD6 emerged as risk mediators, contrasting with the protective effects of CCL3, EIF4EBP1, FGF21, FGF5, and IL7. eGFR analyses delineated opposing regulatory mechanisms (Figure 2B): CCL2, CD274, CX3CL1, CXCL10, DNER, IL12B, NRTN, OSM, STAMBP, SULT1A1, and TGFA decreased eGFR levels, while FGF5, IL18R1, IL20RA, and TRAIL enhanced renal function. Serum creatinine analyses revealed that CCL20, FGF5, IL18R1, IL1A, and TRAIL reduced creatinine accumulation, whereas CCL2, CD274, CXCL10, NRTN, and TGFA exacerbated retention (Figure 2C). Microalbuminuria risk was attenuated by ADA, FGF5, FGF19, and MCSF, yet amplified by CCL11, CXCL1, and FGF21 (Figure 2D). Weighted median and MR-Egger sensitivity analyses corroborated these findings (Supplementary Figure 3), identifying FGF5’s pleiotropic renoprotective effects across all renal endpoints. After Bonferroni correction, DNER (OR=0.995; 95% CI: 0.993–0.998; p=0.001) and NRTN (OR=0.995; 95% CI: 0.992–0.998; p=0.002) decreased the level of eGFR, whereas FGF5 (OR=1.004; 95% CI: 1.002–1.006; p<0.001) and IL18R1 (OR=1.002; 95% CI: 1.001–1.003; p=0.001) increased the level of eGFR. CD274 (OR=1.030; 95% CI: 1.010–1.051; p=0.003) and CXCL10 (OR=1.024; 95% CI: 1.009–1.040; p=0.002) increased the level of serum creatinine, whereas FGF5 (OR=0.975; 95% CI: 0.964–0.987; p<0.001) and IL18R1 (OR=0.989; 95% CI: 0.983–0.996; p=0.002) decreased the level of serum creatinine. FGF21 (OR=1.448; 95% CI: 1.151–1.821; p=0.002) was associated with an increased risk of microalbuminuria.

**Figure 2:** – The annular heat map results of the 91 inflammatory cytokines on CKD traits. CKD: chronic kidney disease; eGFR: estimated glomerular filtration rate.

### Influence of 91 inflammatory cytokines on obesity-traits

Figure 3 delineates cytokine-obesity associations through annular heatmap analysis. Obesity analyses identified CCL4 as a principal obesity risk mediator, with CXCL5 and LIFR demonstrating protective effects (Figure 3A). BMI analyses revealed IL6, IL33, and MMP1 as positive regulators, contrasting with CCL13’s adiposity-limiting role (Figure 3B). WC analyses revealed that IL17C promoted central adiposity, while CCL13, IL1A, and SIRT2 conferred metabolic protection (Figure 3C). Sensitivity analyses via weighted median and MR-Egger methods confirmed robustness across obesity metrics (Supplementary Figure 4), highlighting CCL13’s dual protective effects on both BMI and WC. After Bonferroni correction, no associations remained significant.

**Figure 3:** – The annular heat map results of the 91 inflammatory cytokines on obesity traits. BMI: body mass index; WC: waist circumstance

### Influence of 91 inflammatory cytokines on diabetes traits

Figure 4 delineates cytokine-diabetes interactions through annular heatmap analysis. Diabetes analyses identified ARTN, CD5, CXCL9, and FLT3LG as pathogenic mediators for diabetes risk, contrasted by FGF21 and IL5’s protective effects (Figure 4A). Fasting glucose regulation involved CCL25, CCL7, HGF, IL10RB, IL18R1, and LIFR, demonstrating glycemic control (Figure 4B). HbA1c analyses revealed bidirectional regulation: FGF19, IL7, and TGFB1 elevated glycation levels, while Caspase8, CD5, FGF21, FGF5, IL6, LIFR, SIRT2, SLAM, and TNF exhibited glycemic benefits (Figure 4C). Weighted median and MR-Egger sensitivity analyses validated these associations (Supplementary Figure 5), identifying FGF21 as dual therapeutic potential through diabetes risk reduction and HbA1c lowering. After Bonferroni correction, FGF19 (OR=1.024; 95% CI: 1.008–1.041; p=0.003) was associated with higher HbA1c level, whereas FGF21 (OR=0.951; 95% CI: 0.921–0.981; p=0.001) and LIFR (OR=0.933; 95% CI: 0.898–0.969; p<0.001) were associated with lower HbA1c level.

**Figure 4:** – The annular heat map results of the 91 inflammatory cytokines on diabetes traits. Hb A1c: glycated hemoglobin A1c; T2D: type 2 diabetes.

The overall study design can be found in the Central Figure.

## Discussion

Our multidimensional MR investigation yielded four pivotal advancements in understanding inflammatory regulation of CKM syndrome: 1) systematic delineation of 60 disease-associated cytokines established context-specific therapeutic targets, 2) identification of FGF5 and IL6 as paradoxical regulators with opposing effects across organ systems, and 3) validation of MCSF as a pan-vascular risk amplifier. These findings challenge simplistic pro-/anti-inflammatory dichotomies, revealing instead a complex interplay of cytokine pleiotropy shaped by tissue-specific receptor biology and temporal dynamics.

Our findings necessitate a fundamental reconceptualization of inflammatory biology, transcending the antiquated “pro-inflammatory = pathogenic” dogma by demonstrating that cytokines operate as context-aware biosensors whose functional outputs are dictated by tissue-specific receptor topography and temporal exposure dynamics. The FGF5 paradox exemplifies this paradigm, where FGF5 was identified as a risk factor for CVD, including AF and CHD, a protective factor for CKD (CKD, eGFR, serum creatinine, and microalbuminuria), and HbA1c control. The FGF5 protein is a member of the fibroblast growth factor family that stimulates cell growth and proliferation in multiple cell types, including cardiac myocytes, and has been associated with hypertrophy and angiogenesis in hibernating myocardium.^29^ This apparent contradiction of our study mirrors recent MR evidence showing FGF5’s tissue-specific modulation. MR and colocalization analyses indicated that genetically determined levels of FGF5 were associated with increased risk of CHD (OR=1.08; 95% CI: 1.05-1.11).^30^ Similarly, the cis-pQTL SNP used as a genetic instrument for FGF5 has been linked to CHD as well.^31^ However, leveraging pQTLs of 3032 proteins from 3 large-scale GWAS and corresponding blood- and tissue-specific eQTLs, the gene expression of FGF5 was negatively associated with CKD in kidney cortex samples.^32,33^ Meanwhile, a higher genetically predicted FGF5 concentration was associated with higher eGFR.^33^ These findings caution against systemic FGF pathway modulation, advocating instead for tissue-targeted delivery systems currently under development.^34^ The protective association of IL6 with CHD initially appears discordant with observational studies linking elevated IL6 to cardiovascular risk.^35^ While acute IL6 spikes promote plaque instability, lifelong moderate elevation (as modeled by MR) may induce adaptive vascular remodeling. Hormesis, which is defined as an adaptive response of cells and organisms to a moderate stress, can activate cardioprotective mechanisms.^36^ Our findings are also consistent with the growing appreciation for the differential effects of “good” and “bad” reactive oxygen species in health benefits.^37^

Our study highlights MCSF (encoded by *CSF1*) as a pivotal and consistent amplifier of vascular risk across major atherosclerotic diseases, including CHD, stroke, and PAD. Concordant with our study, the prognostic value of MCSF was independent and complementary to that of C-reactive protein (CRP) in 100 patients with chronic CHD.^38^ MCSF-activated macrophages migrate to the fibrous cap of an atherosclerotic plaque, produce metalloproteinases, and, thus, may cause plaque destabilization.^39^ MCSF also promotes platelet activation,^40^ tissue factor expression,^41^ and the release of the procoagulant cytokine.^42^ Through these actions, MCSF may promote thrombosis and, thus, contribute to the development of acute coronary syndromes and ischemic stroke in patients with atherosclerosis. Targeting MCSF could represent a novel therapeutic strategy for reducing global vascular risk. Preclinical studies have shown that genetic deletion or pharmacological inhibition of MCSF attenuates atherosclerosis in animal models, reducing plaque burden and inflammation.^43-45^ Translating these findings to clinical practice requires caution and further validation.

To provide mechanistic insights, cytokines operate through tissue- and context-specific signaling pathways that shape CKM progression. IL6 signals via classic and trans modes, with classical signaling mediating homeostatic effects, while trans-signaling promotes endothelial activation, monocyte recruitment, and chronic inflammation, activating JAK/STAT3, PI3K/AKT, and MAPK pathways.^46,47^ FGF5 modulates cell proliferation and vascular remodeling in a tissue-dependent manner, explaining its pleiotropic effects. MCSF drives macrophage recruitment, activation, and plaque destabilization, contributing to vascular risk.^48^ These findings highlight that cytokines function as dynamic, context-aware regulators, where temporal exposure and tissue-specific receptor expression determine pathogenic versus protective roles, supporting CKM as a systemic syndrome rather than isolated organ disease.

Although our findings withstand rigorous sensitivity analyses, several methodological considerations warrant acknowledgment. First, the nonlinear dose-response relationships of cytokines remain uncharacterized due to inherent limitations in summary-level MR approaches, precluding assessment of threshold effects or U-shaped associations. Second, our estimates reflect lifelong genetic predisposition to cytokine modulation, which may not directly mirror the therapeutic effects of acute pharmacological cytokine modulation. Third, sex-dimorphic effects could not be ascertained given the predominantly mixed-sex GWAS sources. Fourth, the genetic instruments for inflammatory cytokines were derived exclusively from cohorts of European ancestry, thereby limiting the generalizability of our findings to other ancestral groups.^11^ Fifth, formal MR power calculations were not performed, which may limit the ability to detect modest associations. Future studies with larger sample sizes are needed to validate these exploratory findings. Finally, given the large number of tests performed, some findings may be subject to false-positive bias. While a Bonferroni correction was conducted, the reduced number of significant associations after adjustment highlights the need for cautious interpretation and independent replication.

## Conclusions

This MR atlas redefines inflammatory networks in cardiovascular, kidney, and metabolic diseases, revealing FGF5 and IL6 as paradoxical regulators requiring context-specific modulation, MCSF as a unifying vascular risk amplifier. Our findings challenge simplistic pro-/anti-inflammatory dichotomies, revealing instead a complex interplay of cytokine pleiotropy shaped by tissue-specific receptor biology and temporal dynamics in the CKM era.

## Supplemental Materials

Supplementary Figure 1. The overview of the study design.
